# Compatibility of ciprofloxacin with commercial peritoneal dialysis solutions

**DOI:** 10.1038/s41598-019-42854-y

**Published:** 2019-04-24

**Authors:** Manuel Kussmann, Alexander Ferth, Markus Obermüller, Petra Pichler, Markus Zeitlinger, Martin Wiesholzer, Heinz Burgmann, Wolfgang Poeppl, Gottfried Reznicek

**Affiliations:** 1 Department of Internal Medicine I, Division of Infectious Diseases, and Tropical Medicine, Medical University Vienna, Vienna, Austria; 2 Department of Pharmacognosy, University of Vienna, Vienna, Austria; 3 Department of Internal Medicine I, University Hospital St. Poelten, Karl Landsteiner, University of Health Sciences, St. Poelten, Austria; 4 Department of Clinical Pharmacology, Medical University Vienna, Vienna, Austria; 5 Department of Dermatology and Tropical Medicine, Military Medical Cluster East, Austrian Armed Forces, Vienna, Austria

**Keywords:** Bacterial infection, Peritoneal dialysis

## Abstract

Intraperitoneal administration of antibiotics together with peritoneal dialysis fluids (PDFs) remains the preferable route for treatment of peritoneal dialysis-related peritonitis. For home based therapy, antibiotic-containing PDFs are stored for up to two weeks and warmed up to body-temperature before administration. The present study investigated the compatibility of ciprofloxacin with five commercial PDFs at refrigeration-temperature, room-temperature and body-temperature. Ciprofloxacin concentrations were determined using high-performance liquid chromatography. Drug-diluent stability was evaluated by measurement of pH-values and visual inspection at each sampling point. The antimicrobial activity of ciprofloxacin was assessed by an *E. coli* disk diffusion method. Ciprofloxacin was stable at refrigeration-temperature and body-temperature in all PDFs evaluated over the whole study period of 14 days and 24 hours, respectively. At room-temperature, in contrast, ciprofloxacin demonstrated only limited stability in particular when tested in mixed Physioneal. Except for Physioneal 1.36%, no relevant drug adsorption was observed and the antimicrobial activity of ciprofloxacin was found to be preserved in each PDF at each storage condition investigated. Intraperitoneal ciprofloxacin might be used for inpatient and home based therapy of peritoneal dialysis-related peritonitis and no compensatory dose adjustment is needed when stored for up to two weeks at refrigeration-temperature before use.

## Introduction

Bacterial peritonitis remains the main infectious complication among patients undergoing peritoneal dialysis (PD)^1,2^. The pathogens isolated from patients with peritoneal dialysis related peritonitis (PDRP) comprise gram positive and gram negative bacteria wherefore empiric antimicrobial regimens should cover both, in consideration of local susceptibility data^1,3–5^. Empirical gram negative coverage might be provided by ciprofloxacin which demonstrates satisfactory response rates when used in combination with either cefazolin or vancomycin, for gram positive coverage^6–9^. Furthermore, ciprofloxacin exhibits good antipseudomonal activity^8,9^. With regard to the International Society of Peritoneal Dialysis (ISPD) recommendations the intraperitoneal (IP) route should be preferred for antimicrobial treatment of PDRP due to considerably higher drug concentrations achieved at the target site^1,10^. Furthermore, IP administration of quinolones might improve the microbiological outcome and, thus, the complete cure rates of PDRP due to avoidance of chelation interactions with oral phosphate binders and other concomitantly administered drugs with the use of oral fluoroquinolones^11–13^. For home based therapy of PDRP, patients are routinely supplied with antibiotic containing peritoneal dialysis fluids (PDFs) which are stored for up to two weeks and warmed up to body-temperature directly before administration. Thus, data on drug stability and compatibility over time with commercially available PDFs are prerequisite to ensure adequate peritoneal drug exposure^1,14^. Due to significant differences in regard to the composition of currently used PDFs, drug compatibility should not be extrapolated from one solution to another. Therefore, the present study investigated the compatibility of ciprofloxacin with five different commercial PDFs namely Extraneal, Nutrineal, Physioneal 1.36%, Physioneal 2.27% and Physioneal 3.86%, at various storage conditions.

## Results

### Physical and chemical compatibility testing

Mean concentrations, inhibitory zone diameters and mean pH values of ciprofloxacin over time in five different commercial PDFs are presented in Tables 1–3, respectively.

**Table 1:** Mean concentrations of ciprofloxacin (mg/L) in five different commercially available peritoneal dialysis fluids, at various temperatures and time points. Temp. = temperature; Conc. = concentration; SD = standard deviation; % CTC = percentage of the calculated target concentration; CTC = calculated target concentration (mg/L);

Temp.	Time (h)	Extraneal			Nutrineal			Physioneal 1.36%			Physioneal 2.27%			Physioneal 3.86%		
		Conc. (mg/L)	SD (mg/L)	% CTC	Conc. (mg/L)	SD (mg/L)	% CTC	Conc. (mg/L)	SD (mg/L)	% CTC	Conc. (mg/L)	SD (mg/L)	% CTC	Conc. (mg/L)	SD (mg/L)	% CTC
**6 °C**		***CTC: 47.62***		***100.0***	***CTC: 47.62***		***100.0***	***CTC: 47.62***		***100.0***	***CTC: 47.62***		***100.0***	***CTC: 47.62***		***100.0***
	0	44.917	0.019	**94.3**	46.734	0.015	**98.1**	49.704	0.030	**104.4**	48.669	0.014	**102.2**	48.415	0.016	**101.7**
	24	44.607	0.001	**93.7**	45.729	0.004	**96.0**	48.547	0.007	**101.9**	48.254	0.015	**101.3**	47.862	0.004	**100.5**
	48	44.457	0.003	**93.4**	45.398	0.110	**95.3**	48.136	0.023	**101.1**	48.290	0.019	**101.4**	47.697	0.028	**100.2**
	72	44.048	0.020	**92.5**	45.175	0.012	**94.9**	48.126	0.027	**101.1**	48.132	0.017	**101.1**	47.832	0.023	**100.5**
	168	43.763	0.020	**91.9**	45.176	0.010	**94.9**	48.137	0.011	**101.1**	48.129	0.022	**101.1**	47.699	0.026	**100.2**
	336	43.740	0.020	**91.9**	45.007	0.015	**94.5**	47.694	0.023	**100.2**	47.552	0.010	**99.9**	47.017	0.013	**98.7**
**25 °C**		***CTC: 47.62***		***100.0***	***CTC: 47.62***		***100.0***	***CTC: 47.62***		***100.0***	***CTC: 47.62***		***100.0***	***CTC: 47.62***		***100.0***
	0	46.990	0.000	**98.7**	47.548	0.008	**99.9**	43.476	0.022	**91.3**	45.453	0.022	**95.5**	45.763	0.016	**96.1**
	6	46.270	0.030	**97.2**	47.256	0.012	**99.2**	36.700	0.004	**77.1**	44.902	0.016	**94.3**	44.781	0.012	**94.0**
	24	46.300	0.040	**97.2**	47.285	0.015	**99.3**	35.754	0.018	**75.1**	43.915	0.021	**92.2**	43.936	0.021	**92.3**
	48	46.310	0.030	**97.3**	47.002	0.009	**98.7**	35.605	0.027	**74.8**	43.075	0.021	**90.5**	42.919	0.015	**90.1**
	72	46.310	0.020	**97.3**	47.008	0.028	**98.7**	35.178	0.005	**73.9**	41.927	0.006	**88.1**	42.359	0.010	**89.0**
	168	46.310	0.040	**97.3**	45.613	0.008	**95.8**	33.054	0.029	**69.4**	34.619	0.031	**72.7**	40.936	0.019	**86.0**
	336	42.050	0.090	**88.3**	41.651	0.027	**87.5**	36.993	0.005	**77.7**	32.386	1.467	**68.0**	36.732	0.008	**77.1**
**37 °C**		***CTC: 47.62***		***100.0***	***CTC: 47.62***		***100.0***	***CTC: 47.62***		***100.0***	***CTC: 47.62***		***100.0***	***CTC: 47.62***		***100.0***
	0	47.640	0.403	**100.0**	47.756	0.387	**100.3**	47.525	0.388	**99.8**	47.264	0.289	**99.3**	46.111	0.434	**96.8**
	2	47.557	0.176	**99.9**	47.459	0.188	**99.7**	47.259	0.153	**99.2**	47.026	0.193	**98.8**	46.114	0.417	**96.8**
	4	47.530	0.03	**99.8**	47.283	0.249	**99.3**	47.169	0.058	**99.1**	47.057	0.159	**98.8**	46.162	0.400	**96.9**
	6	47.530	0.127	**99.8**	47.231	0.081	**99.2**	46.686	0.168	**98.0**	47.077	0.149	**98.9**	46.194	0.391	**97.0**
	12	47.780	0.166	**100.3**	47.565	0.014	**99.9**	46.702	0.124	**98.1**	47.009	0.324	**98.7**	46.268	0.460	**97.2**
	24	47.040	0.118	**98.8**	47.183	0.171	**99.1**	46.659	0.087	**98.0**	46.667	0.313	**98.0**	46.216	0.406	**97.1**

**Table 2:** pH values of ciprofloxacin admixed to different commercial peritoneal dialysis fluids at various temperatures and time points.

Temp.	Time (h)	Extraneal		Nutrineal		Physioneal 1.36%		Physioneal 2.27%		Physioneal 3.86%	
		pH	SD	pH	SD	pH	SD	pH	SD	pH	SD
**6 °C**	0	5.46	0.00	6.57	0.00	7.41	0.00	7.60	0.00	7.48	0.00
	24	5.30	0.00	6.44	0.00	7.74	0.00	7.84	0.00	7.81	0.00
	48	5.33	0.00	6.48	0.00	7.95	0.00	7.97	0.00	7.93	0.00
	72	5.34	0.00	6.60	0.00	7.74	0.00	7.83	0.00	7.82	0.00
	168	5.40	0.00	6.41	0.00	7.69	0.00	7.98	0.00	7.99	0.00
	336	5.62	0.00	6.71	0.00	7.72	0.00	7.97	0.00	7.97	0.00
**25 °C**	0	5.30	0.06	6.51	0.02	7.52	0.00	8.13	0.00	7.76	0.00
	6	5.26	0.01	6.52	0.00	7.59	0.00	8.05	0.00	7.69	0.00
	24	5.28	0.01	6.56	0.01	7.73	0.00	8.19	0.00	7.84	0.00
	48	5.27	0.01	6.55	0.01	8.16	0.00	8.23	0.00	8.22	0.00
	72	5.28	0.01	6.55	0.01	8.13	0.00	8.30	0.00	8.22	0.00
	168	5.30	0.01	6.55	0.01	8.22	0.00	8.42	0.00	8.23	0.00
	336	5.32	0.00	6.55	0.00	8.28	0.00	8.37	0.00	8.31	0.00
**37 °C**	0	5.28	0.01	6.65	0.01	7.87	0.02	8.02	0.31	8.15	0.01
	1	5.27	0.01	6.72	0.02	7.78	0.04	7.89	0.39	7.88	0.02
	2	5.25	0.01	6.75	0.02	7.75	0.01	7.91	0.38	8.16	0.01
	4	5.26	0.01	6.68	0.03	7.87	0.01	7.88	0.32	7.83	0.01
	12	5.27	0.02	6.70	0.03	7.79	0.01	7.96	0.38	7.77	0.01
	24	5.26	0.01	6.82	0.03	8.01	0.03	8.14	0.25	8.22	0.03

**Table 3:** Inhibitory zone diameters of the *Escherichia coli* inhibition assay in % of the calculated target concentrations (% IZD) in different commercial peritoneal dialysis fluids at various temperatures and time points. Temp. = temperature; %RSD = percentage relative standard deviation.

Temp.	Time (h)	Extraneal		Nutrineal		Physioneal 1.36%		Physioneal 2.27%		Physioneal 3.86%	
		%IZD	%RSD	%IZD	%RSD	%IZD	%RSD	%IZD	%RSD	%IZD	%RSD
**6 °C**	0	98.5	0.0	102.3	1.1	104.6	0.0	102.3	1.1	99.2	1.1
	6	99.2	1.1	99.2	1.1	103.8	1.1	102.3	1.1	100.0	0.0
	24	100.8	1.1	105.4	1.1	103.1	0.0	103.1	0.0	100.0	0.0
	48	96.2	1.1	100.8	1.1	102.3	1.1	103.8	1.1	100.0	0.0
	72	96.9	2.2	100.8	1.1	103.1	0.0	103.1	0.0	100.0	0.0
	168	97.7	1.1	101.5	0.0	103.1	0.0	102.3	1.1	100.8	1.1
	336	100.4	0.5	100.8	1.1	100.0	0.0	103.1	0.0	100.8	1.1
**25 °C**	0	104.6	0.0	102.3	1.1	100.8	1.1	102.3	1.1	103.8	1.1
	6	106.9	1.1	101.5	0.0	100.0	0.0	102.3	1.1	103.8	1.1
	24	100.8	1.1	100.8	1.1	100.8	1.1	100.0	0.0	102.3	1.1
	48	105.4	1.1	102.3	1.1	100.8	1.1	100.0	0.0	98.5	0.0
	72	104.6	0.0	103.1	0.0	100.8	1.1	96.9	0.0	97.7	1.1
	168	104.6	0.0	100.8	1.1	101.5	0.0	97.7	1.1	96.9	0.0
	336	105.4	1.1	99.2	1.1	101.5	0.0	93.1	1.1	99.2	1.1
**37 °C**	0	97.7	1.1	101.5	0.0	103.8	1.1	100.0	0.0	100.0	0.0
	1	97.7	1.1	100.8	1.1	104.6	0.0	99.2	1.1	101.5	0.0
	2	98.5	0.0	101.5	0.0	103.1	0.0	99.2	1.1	101.5	0.0
	4	98.5	0.0	99.2	1.1	102.3	1.1	99.2	1.1	99.2	1.1
	12	97.7	1.1	98.5	2.2	100.8	1.1	98.5	0.0	97.7	1.1
	24	103.8	1.1	100.8	1.1	103.8	1.1	99.2	1.1	100.8	1.1

Defined as the time from preparation until the original concentration is reduced to $\le$ 90%, ciprofloxacin was stable in all PDFs over the whole study period of 14 days at refrigeration-temperature (6 °C) and 24 hours at body-temperature (37 °C). At room-temperature (25 °C), in contrast, ciprofloxacin was stable over 7 days in Extraneal, over 2 days in mixed Physioneal 2.27% and Physioneal 3.86%, and less than 6 hours in mixed Physioneal 1.36% (Table 1). In aqueous solution controls, ciprofloxacin was stable at each storage condition over the whole study period with concentrations ranging between 97.9–103.6% of the calculated target concentration.

Visual inspection revealed no color changes, particulate matter or haze over the whole study period in any PDF, at any storage condition investigated. Significantly increased pH values were observed for all PDFs stored at refrigeration-temperature and for all PDFs except Extraneal when stored at room-temperature (for all p < 0.01). At body-temperature, significant pH increases were observed for Nutrineal, Physioneal 1.36% and Physioneal 3.86% (Table 2). With a maximum difference of 8.7% between the calculated target concentration and the initially measured concentration in Physioneal 1.36%, no relevant initial drug adsorption was observed in any PDF at any storage temperature evaluated (Table 1).

### *In vitro* antimicrobial activity testing

The antimicrobial activity of ciprofloxacin was evaluated by an *E. coli* diffusion disk inhibition assay which demonstrated relative inhibitory zone diameters ranging from 93.1 to 106.9% of the calculated target concentration in aqueous solution. Thus, no relevant reduction of antimicrobial activity was observed over the whole study period in any PDF and at any storage condition investigated (Table 3). PDFs without study drug demonstrated no antimicrobial activity (data not shown).

## Discussion

Intraperitoneal ceftazidime, cefepime or aminoglycosides are currently recommended first line therapies for gram negative coverage in empiric treatment of PDRP. However, several clinical studies demonstrated that antimicrobial regimens containing oral fluoroquinolones for gram negative coverage achieve comparable results^6,7,15^.

Pharmacokinetic studies have shown that IP administration of ciprofloxacin results in a markedly higher IP drug exposure, in particular among PD patients with frequently prescribed co-medications like phosphate-binding aluminum antacids which significantly reduce bioavailability of oral ciprofloxacin^16–18^.

As a fluoroquinolone antimicrobial, ciprofloxacin exhibits concentration-dependent killing with Cmax/MIC or AUC/MIC as the best fitting PK/PD indices^19^. With regard to these indices, a Cmax/MIC > 10 or AUC/MIC > 125 (for gram-negative pathogens) was associated with improved clinical and microbiological outcome^19^. Thus, IP administration of ciprofloxacin might be beneficial for patients with PDRP.

Noteworthy, a recently published study investigated the influence of differently composited PDFs on the *in vitro* activity of cefepime, ciprofloxacin, ertapenem, meropenem and tobramycin^20^. It was demonstrated that the activity of time-dependent antimicrobials such as cefepime, ertapenem and meropenem was strongly reduced whereas the concentration-dependent drugs ciprofloxacin and tobramycin were highly active and demonstrated dose-dependent bactericidal activity in all PDFs investigated^20^. Thus, the markedly higher ciprofloxacin concentrations achieved over IP route, together with the avoidance of chelation interactions of concomitantly administered fluoroquinolones, might improve clinical and microbiological outcomes of patients with PDRP.

However, compatibility data with commercially available PDFs are prerequisite for clinical usage of IP ciprofloxacin, but to date there are only two studies available which revealed partly discrepant results. Mawhinney *et al*. investigated the stability of ciprofloxacin 25 mg/L in 1.36% glucose-containing PDFs over 42 days at 4 °C, 20 °C and 37 °C^21^. In that study, ciprofloxacin was stable at each storage condition over the whole study period^21^. Kane *et al*. evaluated the stability of ciprofloxacin 25 mg/L in 1.5% and 4.25% glucose-containing PDFs over 2 weeks at 4 °C, one week at 25 °C and 2 days at 37 °C^22^. Sufficient stability of ciprofloxacin was observed only at 25 °C and 37 °C, whereas at 4 °C drug concentrations were reduced marginally below 90% within the first time interval of 12 hours^22^. Due to the rapid initial decline and the lack of a further decrease in drug concentrations between hours 12 and 336 drug adsorption to the container material was concluded by the authors to be the most probable explanation^22^. In both studies, drug concentrations were determined using high-performance liquid chromatography (HPLC). However, in these studies no differently composited commercial PDFs such as amino acid or icodextrin-containing solutions, which are frequently used in clinical routine, were investigated. Further, according to the recommendations by De Vin *et al*., currently used PDFs possess significant differences in their composition (e.g. buffer, osmotic agent, pH) and therefore compatibility data should not be extrapolated from one PDF or antibiotic to another^14^.

The present study investigated the compatibility of ciprofloxacin with five different commercial PDFs at refrigeration-temperature (6 °C), room-temperature (25 °C) and body-temperature (37 °C). Ciprofloxacin was stable in all PDFs, over the whole respective study period of 24 hours at body-temperature (37 °C) and 14 days at refrigeration-temperature (6 °C), but only over 7 days in Extraneal, < 6 hours in mixed Physioneal 1.36% and 2 days in mixed Physioneal 2.27% and Physioneal 3.86%, when tested at room-temperature (25 °C).

Interestingly, ciprofloxacin was stable in Physioneal 1.36% at 6 °C and 37 °C but not at 25 °C demonstrating an initial drop after the first measurement without a further decline over time. A previous study by Manley *et al*. investigated the stability of amphotericin B lipid complex (ABLC) in PDFs at three different temperatures (4 °C, 25 °C and 37 °C). When evaluated in 1.5% glucose containing PDF at 4 °C and 37 °C ABLC was stable over the whole study period whereas at 25 °C, ABLC concentrations dropped below 80% at the first time point with a relatively little further decline, suggesting drug adsorption to the PVC container material not decomposition^23^.

Significantly increased pH values were observed in diverse PDFs at all storage conditions investigated. However, these pH changes have not influenced chromatographic results and drug degradation > 10% was only observed at room-temperature (25 °C). Nonetheless, the activity of commonly used antimicrobials like aminoglycosides or some beta-lactams might be influenced by pH changes and should therefore be taken into account for combination therapy^24–27^. However, for the first generation cephalosporin cefazolin and the glycopeptide vancomycin, both first-line antimicrobials for gram-positive coverage of PDRP, no significant differences of antimicrobial activity were observed under varying pH conditions^25,26^. Thus, ciprofloxacin in combination with cefazolin or vancomycin might be a useful alternative for empiric treatment of PDRP.

With regard to diluent stability and drug-container interactions, visual inspection of drug containing and control PDFs revealed no abnormalities and, further, no relevant initial drug adsorption was observed in any PDF at any storage condition investigated. Aside from chemical and physical stability, the antimicrobial activity of ciprofloxacin was found to be preserved as demonstrated by the *E. coli* disk diffusion assay.

The following limitations of this study should be noted. First, all experiments were performed with peritoneal dialysis solutions of only one manufacturer. Second, the compatibility of ciprofloxacin with different commercial PDFs was only investigated alone.

Thus, if ciprofloxacin is used with similar PD solutions of other brands or in combination with concomitantly administered drugs such as antibiotics, heparin or insulin, potential influences on its compatibility should be taken into account.

In conclusion ciprofloxacin was stable at refrigeration-temperature (6 °C) and body-temperature (37 °C) in all PDFs evaluated over the whole study period of 14 days and 24 hours, respectively. At room-temperature (25 °C), in contrast, ciprofloxacin demonstrated only limited stability in particular when tested in mixed Physioneal. Therefore, ciprofloxacin might be used for inpatient and home based therapy of PDRP and no compensatory dose adjustment is needed when stored at refrigeration-temperature for up to two weeks before usage. Further studies are warranted to investigate the clinical efficacy of IP ciprofloxacin.

## Methods

### Sample preparation

The compatibility of ciprofloxacin with five different PDFs (Extraneal 2000 mL, 75 g/L icodextrin; Nutrineal 2000 mL, 1.1% amino acids; Physioneal 40 1.36% glucose 2000 mL (chamber A 725 mL, chamber B 1275 mL); Physioneal 40 2.27% glucose 2000 mL (chamber A 725 mL, chamber B 1275 mL) and Physioneal 40 3.86% glucose 2000 mL (chamber A 725 mL, chamber B 1275 mL); all Baxter Healthcare Corp., Deerfield, IL, USA) was investigated at three different storage conditions: over 14 days at refrigeration-temperature (6 °C) and room-temperature (25 °C) and over 24 hours at body-temperature (37 °C). Ciprofloxacin (Ciprofloxacin Kabi 400 mg/200 mL, Meiji Seika Pharma Co. Ltd.) was obtained in form of 2 mg/mL infusion solutions. Fifty milliliters of the ciprofloxacin solution were measured with a volumetric flask and injected into the respective PDF bags sterilely. To minimize residues, the volumetric flask was washed out with 50 mL aqua bidest. and the obtained solution was injected additionally. For the two-chambered bag system of Physioneal, ciprofloxacin was injected into the low-pH chamber A (725 mL) which was mixed with the buffer containing chamber B (1275 mL) directly after administration. Thus, the calculated ciprofloxacin concentration in each PDF investigated was 47.62 mg/L. Respectively, three ciprofloxacin containing PDF bags plus one control PDF bag without study drug were used at each storage condition investigated. Thus, in total, 60 PDF bags were used in the present study. Additively, glass containers with equal ciprofloxacin concentrations in aqueous solutions were run as control at each storage condition investigated. All PDF bags and glass containers were stored light-protected in temperature-controlled rooms and sampling was performed in duplicates as follows: 0 (directly after injection of ciprofloxacin), 24, 48, 72, 168, 336 hours for storage at refrigeration-temperature (6 °C); 0, 6, 24, 48, 72, 168, 336 hours at room-temperature (25 °C) and 0, 2, 4, 6, 12, 24 hours at body-temperature (37 °C). Ciprofloxacin concentrations were determined using high-performance liquid chromatography (HPLC) and drug stability was defined as the time from preparation until the original content is reduced to $\le$ 90% by chemical degradation.

### *In vitro* antimicrobial activity testing

A disk diffusion inhibition assay with *Escherichia coli* (ATCC 25922) was used to evaluate the antimicrobial activity of ciprofloxacin after exposure to different storage conditions and periods. Therefore, bacteria were grown overnight on Columbia agar plates (COS; 5% sheep blood Columbia agar plates, Biomerieux), resuspended in 0.9% sterile saline to a concentration equivalent to 0.5 McFarland standard and plated on COS agar plates. Each sampling point was evaluated in duplicates by impregnating 6 mm filter disks with the respective sample. Inhibitory zone diameters were measured after an incubation of 24 hours at 37 °C ($\pm$ 1 °C). Obtained inhibitory zone diameters were compared to the initially measured concentrations, directly after injection of ciprofloxacin, and are presented in Table 2. Aqueous solutions containing equal ciprofloxacin concentrations and blank PDFs without ciprofloxacin were tested for quality assurance (data not shown). For assessment of drug adsorption (drug-container interactions) calculated target concentrations were compared to the initially measured concentrations obtained directly after injection, as recommended by De Vin *et al*.^14^. To evaluate drug-diluent stability, the pH was measured in duplicates and all PDF bags were visually inspected for haze, particulate matter or color changes, at each sampling point.

### Sample Analysis by High Performance Liquid Chromatography (HPLC)

For the chemical analysis of ciprofloxacin stability, an HPLC-system (Shimadzu LC-20 series) was employed consisting of a DGU-20A5 degasser, a LC-20AD quaternary pump, a SIL-20AC autosampler, a CBM-20A communication bus module, a SPD-M20A diode array detector and a CTO-20AC column oven, operated via Shimadzu LabSolutions software 5.57 SP1. LC separation was performed on an Acclaim Polar Advantage II C18 column (3 µm, 120 Å, 150 × 2.1 mm I.D., Thermo Fisher Scientific), preceded by an Acclaim Polar Advantage II C18 guard cartridge (5 µm, 120 Å, 10 × 2 mm I.D., Thermo Fisher Scientific), at a flow rate of 0.5 mL/min and a column temperature of 25 °C. The mobile phase consisted of a linear gradient mixed from 0.1% aqueous formic acid (mobile phase A) and acetonitrile with 0.1% formic acid (mobile phase B). The linear gradient started from 1% B to 44% B in 10 min, then purging with 95% B for 5 min., and again 1% B to equilibrate the column for 5 min before application of the next sample (total analysis time 20 min). The injection volume was 5 µl each and the detection wavelength was set at 275 nm to quantify peak areas of standards, samples and possible degradation products, ciprofloxacin eluted at 7.74 min. No shift of the retention time could be observed at all during the complete period of analysis. The quantification was carried out using a calibration curve with ciprofloxacin as external standard. After collection the samples were analyzed immediately. The autosampler tray was kept at 10 °C, preliminary investigations showed that the storage of the samples in the autosampler at 10 °C till analysis did not affect the ciprofloxacin concentrations at all (data not shown). All sample series were analyzed within one day, at each timepoint samples from 3 PD bags were taken and analyzed in duplicate (6 measurements per timepoint and temperature) and the mean value was calculated. Each sample series was interspersed with several Quality Control (QC) samples of known concentrations to ensure the validity of the results. Ciprofloxacin gave an isolated peak in the chromatogram with nice peak shape (symmetry factor 1.0 to 1.15) and no generated degradation products were detected in any of the sample solutions. All chromatograms were re-evaluated via PDA using other non-selective wavelength (e.g. 190 nm), no further peaks, peak shoulders or similar phenomena could be observed in comparison to PDF without drug, indicating no coeluting other compound(s). The peak purity of the ciprofloxacin peak was checked for all analyses via PDA, peak purities of 0.9999 were found for the ciprofloxacin peaks (range from 0 to 1.0000) attesting no coeluting degradation products. System suitability test of the standard solution gave 0.47% RSD (peak area, *n* = 6). To test the specificity of the method, the single PDFs were injected and no peak could be detected within the detection window of ciprofloxacin. No carry over could be seen after consecutive injection (10 times) of the standard solution. The precision of the method (ciprofloxacin solution in PDF) gave 0.79% RSD (peak area, *n* = 6). The linearity of the standard solution between 0,5 mg/L and 150 mg/L (1% to 300% of the expected ciprofloxacin concentration) showed *r* = 0.9998. The concentrations are provided as mean values (3 PDF bags each analysed twice at the same time point and temperature).

### Ethical approval

This article does not contain any studies with human participants or animals performed by any of the authors.

## Data Availability

The datasets generated during and/or analysed during the current study are available from the corresponding author on reasonable request.

## References

[1] Li PK-T (2016). ISPD Peritonitis Recommendations: 2016 Update on Prevention and Treatment. Perit. Dial. Int..

[2] Davenport A (2009). Peritonitis remains the major clinical complication of peritoneal dialysis: the London, UK, peritonitis audit 2002-2003. Perit. Dial. Int..

[3] van Esch S, Krediet RT, Struijk DG (2014). 32 years’ experience of peritoneal dialysis-related peritonitis in a university hospital. Perit. Dial. Int..

[4] Huang S-T (2011). Evolution of microbiological trends and treatment outcomes in peritoneal dialysis-related peritonitis. Clin. Nephrol..

[5] Zelenitsky SA (2017). Microbiological Trends and Antimicrobial Resistance in Peritoneal Dialysis-Related Peritonitis, 2005 to 2014. Perit. Dial. Int..

[6] Lima RCS, Barreira A, Cardoso FL, Lima MHS, Leite M (2007). Ciprofloxacin and cefazolin as a combination for empirical initial therapy of peritoneal dialysis-related peritonitis: five-year follow-up. Perit. Dial. Int..

[7] Goffin E (2004). Vancomycin and ciprofloxacin: systemic antibiotic administration for peritoneal dialysis-associated peritonitis. Perit. Dial. Int..

[8] Bauernfeind A, Petermüller C (1983). *In vitro* activity of ciprofloxacin, norfloxacin and nalidixic acid. Eur. J. Clin. Microbiol..

[9] Follath F (1986). Use of ciprofloxacin in the treatment of Pseudomonas aeruginosa infections. Eur. J. Clin. Microbiol..

[10] Gorman T, Eisele G, Bailie GR (1995). Intraperitoneal antibiotics effectively treat non-dialysis-related infections. Perit. Dial. Int..

[11] How PP, Fischer JH, Arruda JA, Lau AH (2007). Effects of lanthanum carbonate on the absorption and oral bioavailability of ciprofloxacin. Clin. J. Am. Soc. Nephrol..

[12] Kays MB, Overholser BR, Mueller BA, Moe SM, Sowinski KM (2003). Effects of sevelamer hydrochloride and calcium acetate on the oral bioavailability of ciprofloxacin. Am. J. Kidney Dis..

[13] Cheng IK (1993). A randomized prospective comparison of oral versus intraperitoneal ciprofloxacin as the primary treatment of peritonitis complicating continuous ambulatory peritoneal dialysis. Perit. Dial. Int..

[14] de Vin F, Rutherford P, Faict D (2009). Intraperitoneal administration of drugs in peritoneal dialysis patients: a review of compatibility and guidance for clinical use. Perit. Dial. Int..

[15] Cheng IK (1998). A randomized prospective comparison of oral levofloxacin plus intraperitoneal (IP) vancomycin and IP netromycin plus IP vancomycin as primary treatment of peritonitis complicating CAPD. Perit. Dial. Int..

[16] Yeung SM (2004). Pharmacokinetics of oral ciprofloxacin in continuous cycling peritoneal dialysis. Perit. Dial. Int..

[17] Golper TA, Hartstein AI, Morthland VH, Christensen JM (1987). Effects of antacids and dialysate dwell times on multiple-dose pharmacokinetics of oral ciprofloxacin in patients on continuous ambulatory peritoneal dialysis. Antimicrob. Agents Chemother..

[18] Ludlam HA (1990). Intraperitoneal ciprofloxacin for the treatment of peritonitis in patients receiving continuous ambulatory peritoneal dialysis (CAPD). J. Antimicrob. Chemother..

[19] Scaglione F, Paraboni L (2006). Influence of pharmacokinetics/pharmacodynamics of antibacterials in their dosing regimen selection. Expert Rev. Anti Infect. Ther..

[20] Kussmann M (2016). Influence of Different Peritoneal Dialysis Fluids on the *In Vitro* Activity of Cefepime, Ciprofloxacin, Ertapenem, Meropenem and Tobramycin Against Escherichia Coli. Perit. Dial. Int..

[21] Mawhinney WM, Adair CG, Gorman SP, McClurg B (1992). Stability of ciprofloxacin in peritoneal dialysis solution. Am. J. Hosp. Pharm..

[22] Kane MP, Bailie GR, Moon DG, Siu I (1994). Stability of ciprofloxacin injection in peritoneal dialysis solutions. Am. J. Hosp. Pharm..

[23] Manley HJ (2000). Stability of amphotericin B-lipid complex (Abelcet) in peritoneal dialysis solutions. Perit. Dial. Int..

[24] Burian A, Erdogan Z, Jandrisits C, Zeitlinger M (2012). Impact of pH on activity of trimethoprim, fosfomycin, amikacin, colistin and ertapenem in human urine. Pharmacology.

[25] Lamp KC, Rybak MJ, Bailey EM, Kaatz GW (1992). *In vitro* pharmacodynamic effects of concentration, pH, and growth phase on serum bactericidal activities of daptomycin and vancomycin. Antimicrob. Agents Chemother..

[26] König C, Simmen HP, Blaser J (1993). Effect of pathological changes of pH, pO2 and pCO2 on the activity of antimicrobial agents *in vitro*. Eur. J. Clin. Microbiol. Infect. Dis..

[27] Schlessinger D (1988). Failure of aminoglycoside antibiotics to kill anaerobic, low-pH, and resistant cultures. Clin. Microbiol. Rev..

